# Supplementary figures and images for: Extracellular vesicles from adipose-derived mesenchymal stem cells promote colony formation ability and EMT of corneal limbal epithelial cells
Source: PLoS One. 2025 Apr 21;20(4):e0321579. doi: 10.1371/journal.pone.0321579 (PMC12011229; doi:10.1371/journal.pone.0321579)

# S1 Fig

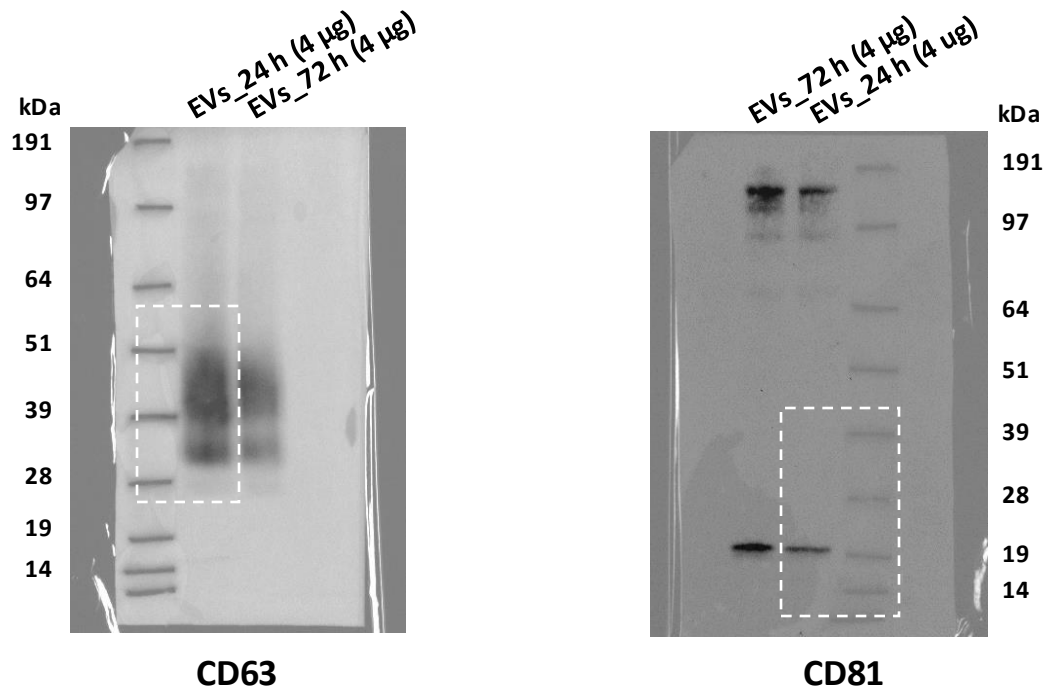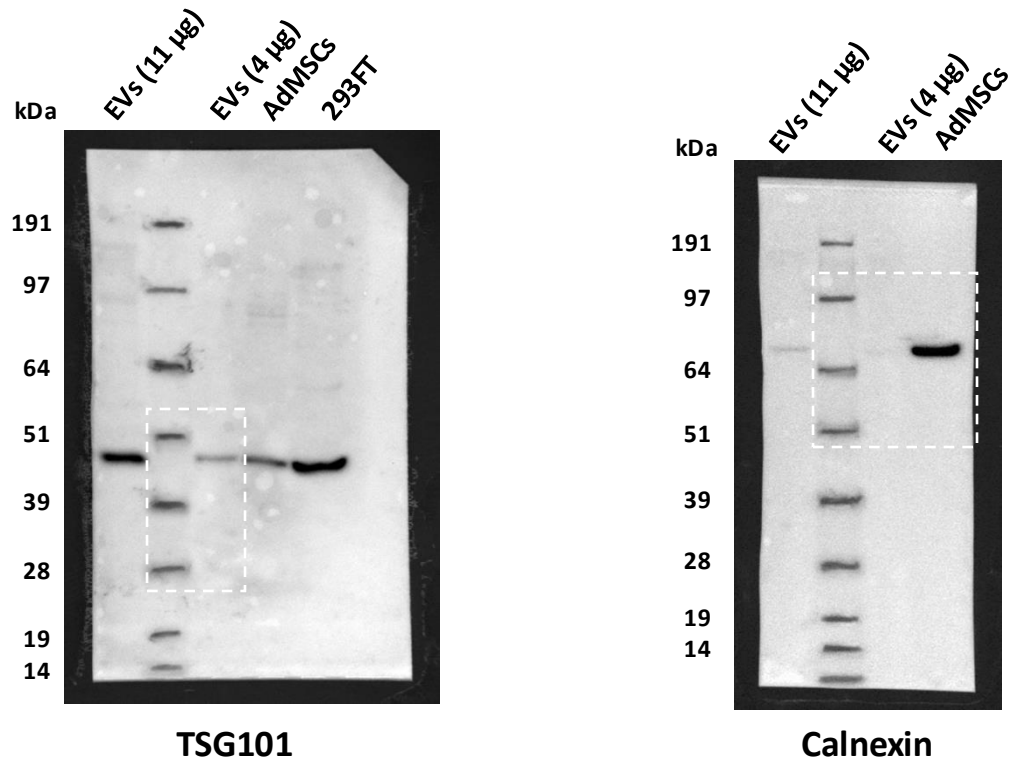

Supplement: S1 Fig — Original images of CD63 (30–60 kDa), CD81 (~25 kDa), TSG101 (~46 kDa) and calnexin (~78 kDa) analysed by western blotting. EVs_24 h/72 h: EVs were isolated from the conditioned medium that were cultured for 24 h/72 h. AdMSCs and 293FT cells were used as positive controls. White dashed rectangles show the cropped part used in Fig 1C. (PDF) [file pone.0321579.s001.pdf]

# S2 Fig

**A**

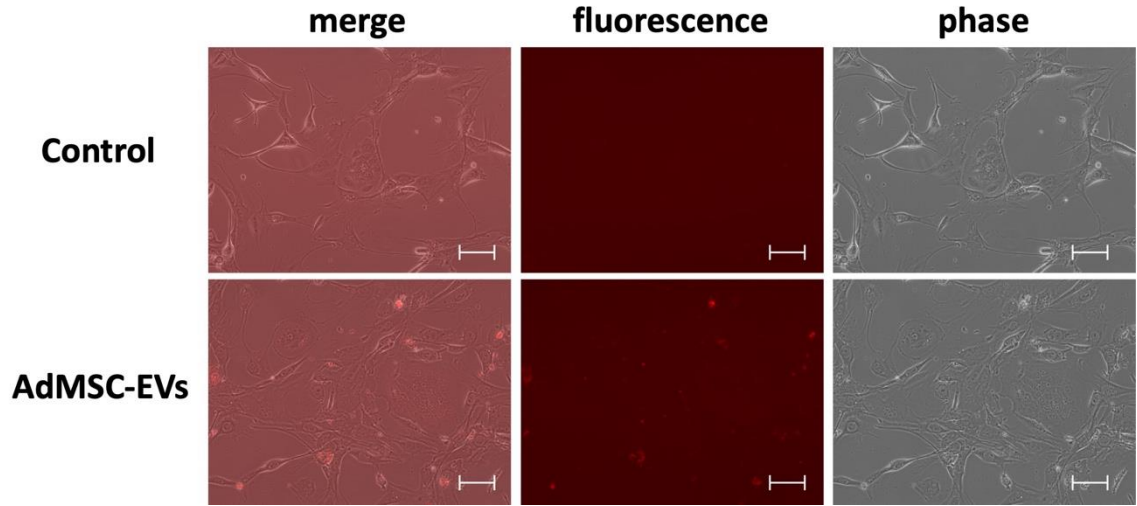

**B**

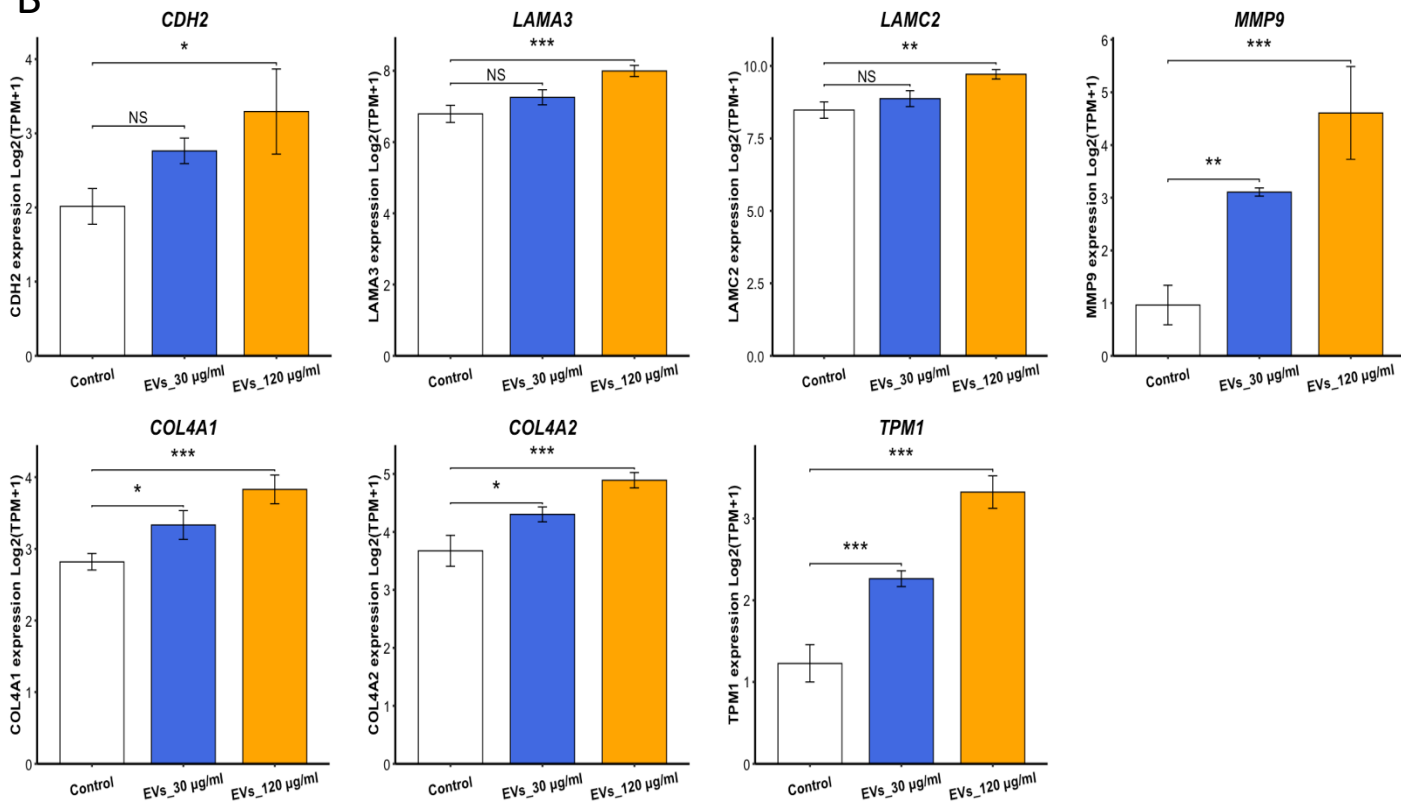

**C**

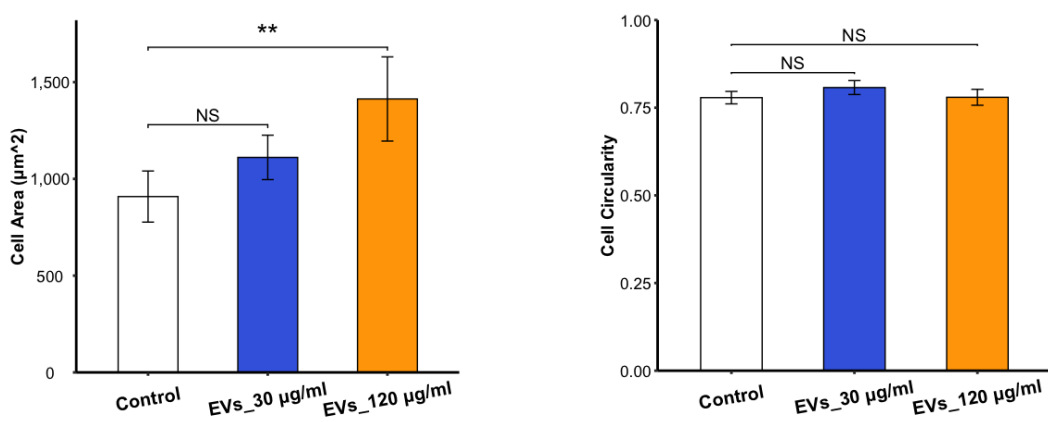

Supplement: S2 Fig — (A) Images of the extracellular vesicles from human adipose-derived mesenchymal stem cells (AdMSC-EVs) labelled with Mem Dye-Red during the colony formation assay in the phosphate-buffered saline (PBS) control group and EV-treated group. (B) Comparisons of expression levels of additional genes related to epithelial-mesenchymal transition (EMT) by RNA-Seq analysis between the control and EV-treated groups. The results are shown as the mean ± SD. n = 3 biological replicates. (C) Cell morphological analysis of EV-treated LECs. The results are expressed as the mean ± SD. n = 4 biological replicates. NS: not significant. *p < 0.05, **p < 0.01, and ***p < 0.001. (PDF) [file pone.0321579.s002.pdf]

S3 Fig

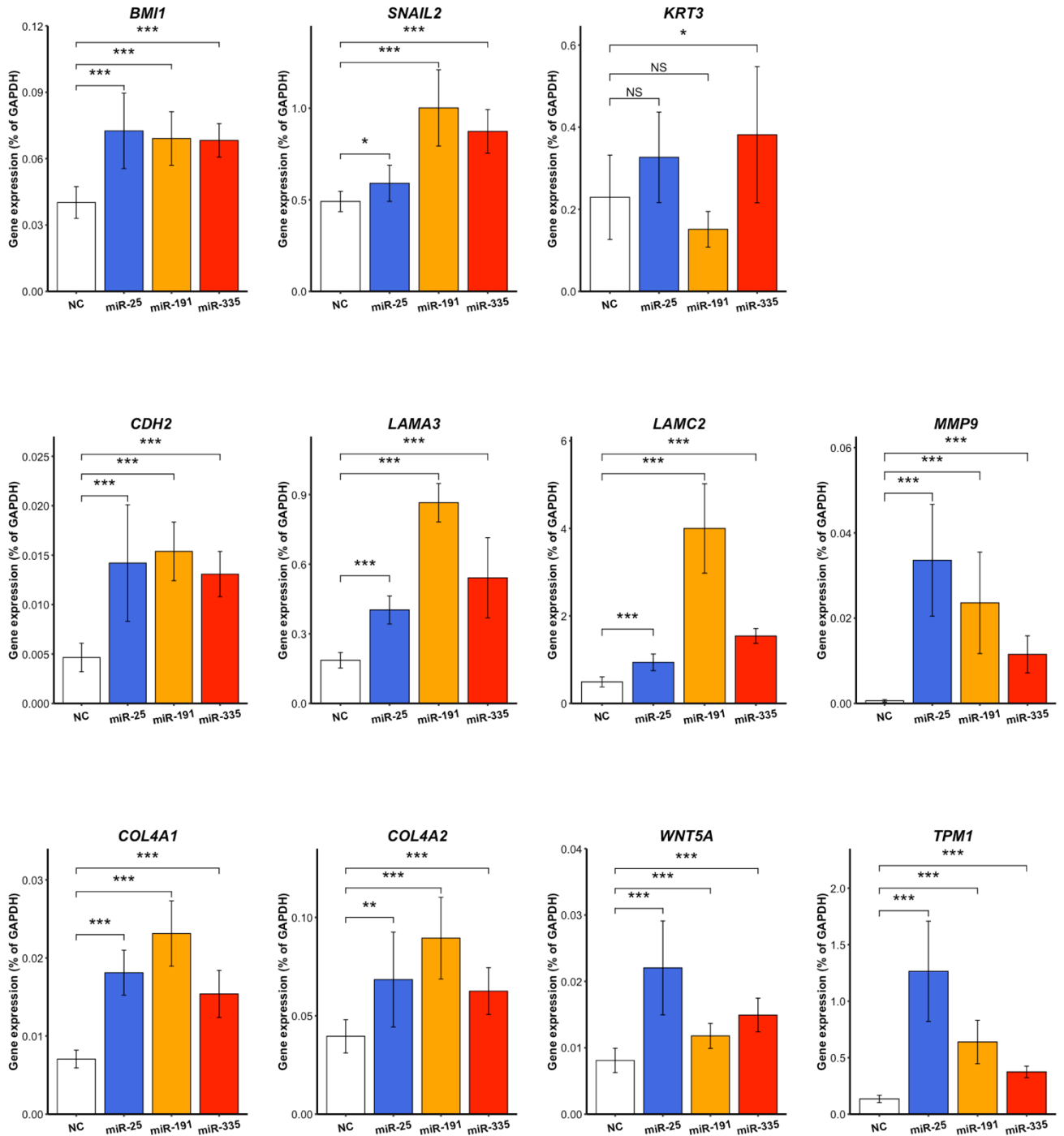

Supplement: S3 Fig — Quantitative reverse transcription-polymerase chain reaction analysis of genes related to stemness (BMI1, SNAIL2), differentiation (KRT3) and epithelial-mesenchymal transition (CDH2, LAMA3, LAMC2, MMP9, COL4A1, COL4A2, WNT5A, TPM1), compared between the NC group and microRNA (miRNA)-transfected groups. The results are expressed as the mean ± SD. n =8 biological replicates. (PDF) [file pone.0321579.s003.pdf]

# S4 Fig

A

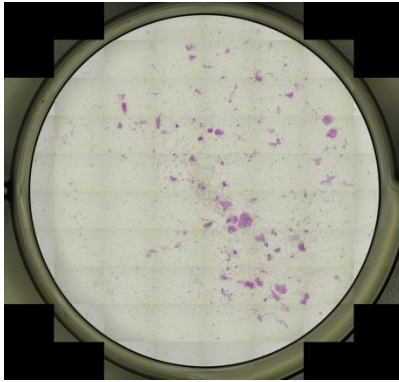

NC

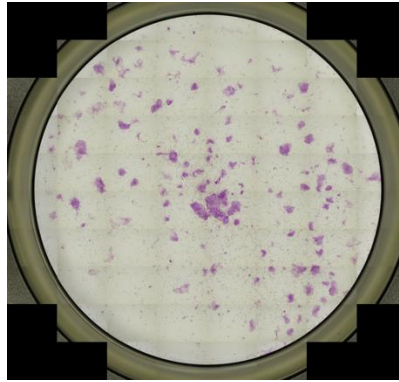

Mix(3)

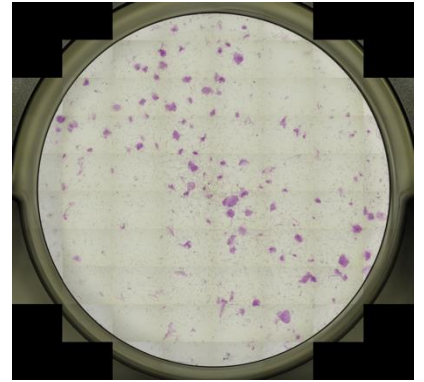

Mix(6)

B

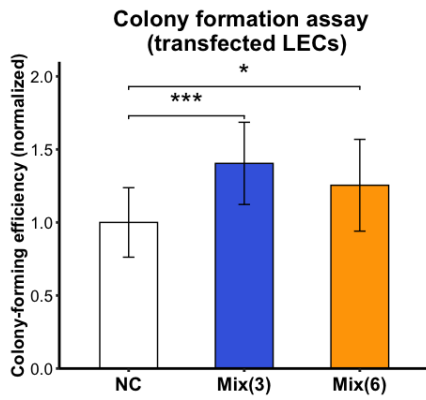

C

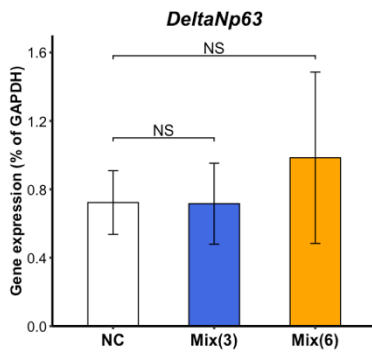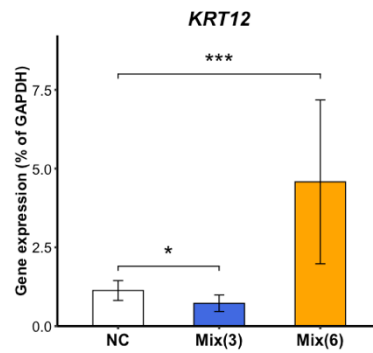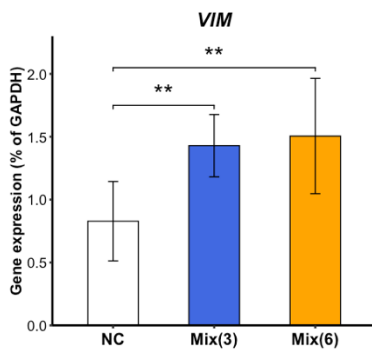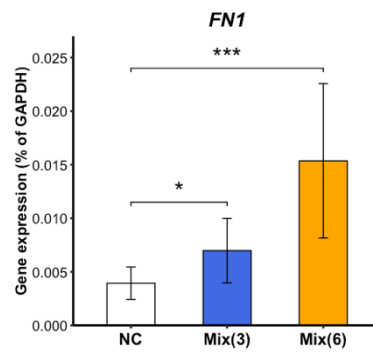

Supplement: S4 Fig — (A) Images showing colonies of the transfected LECs. n = 16 biological replicates. (B) The colony-forming efficiency of LECs from the NC group and miRNA mixture groups, presented as the mean ± standard deviation (SD). n = 16 biological replicates. (C) Quantitative reverse transcription-polymerase chain reaction analysis of TP63, KRT12, VIM, and FN1 between the NC and miRNA mixture groups. n =7 biological replicates. NS: not significant. *p < 0.05, **p < 0.01, and ***p < 0.001. NC: negative control. Mix(3): mixture of miR-25, miR-191 and miR-335. Mix(6): mixture of miR-25, miR-126, miR-130, miR-191, miR-223 and miR-335. (PDF) [file pone.0321579.s004.pdf]

**S5 Fig**

**A**

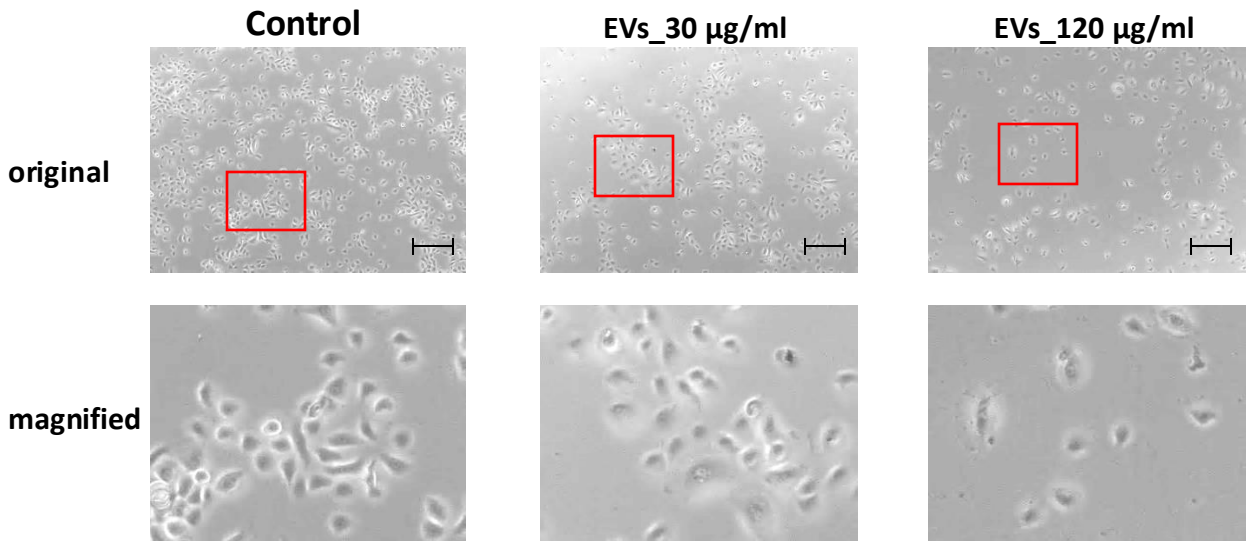

**B**

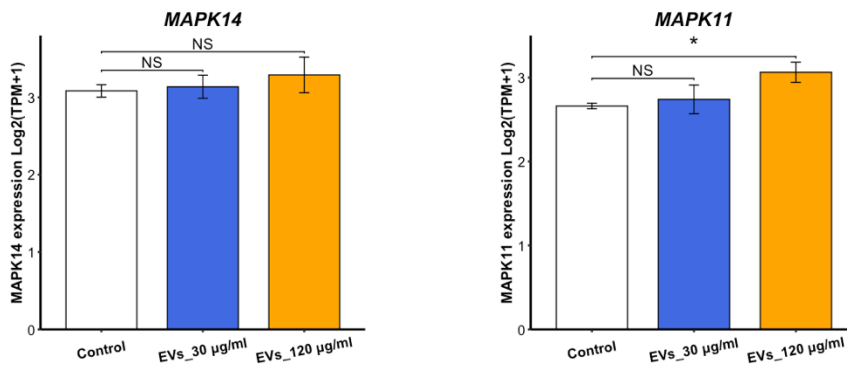

Supplement: S5 Fig — (A) Original and magnified images of LECs after 72 h in the control and EV-treated groups. Red rectangles in the original images show the magnified part. (B) Comparisons of expression levels of genes encoding p38α (MAPK14) and p38β (MAPK11) between the control and EV-treated groups from RNA-Seq data. The results are shown as the mean ± SD. n = 3 biological replicates. NS: not significant. *p < 0.05, **p < 0.01, and ***p < 0.001. NC: negative control. (PDF) [file pone.0321579.s005.pdf]

**S6 Fig**

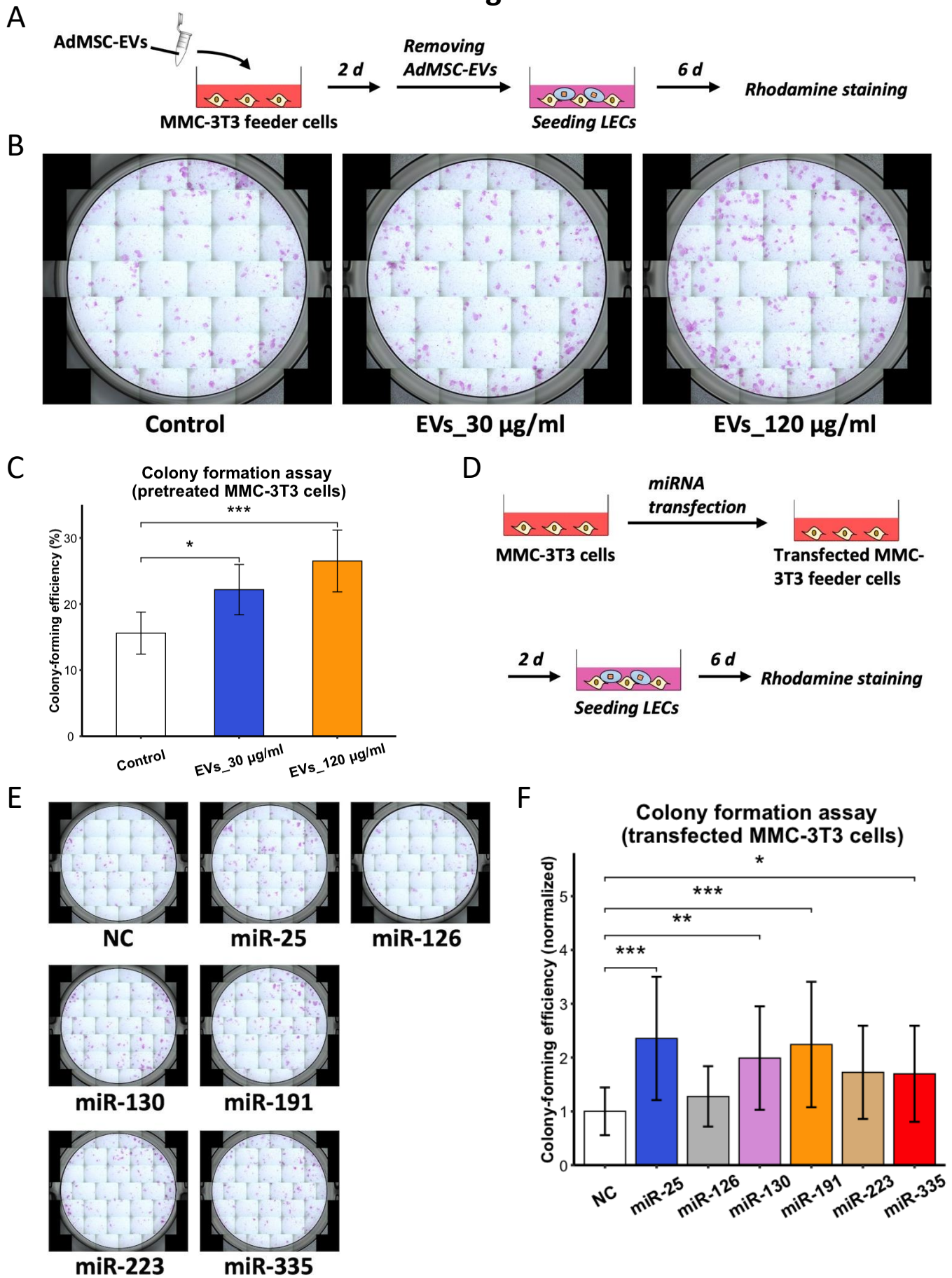

Supplement: S6 Fig — (A) Methodology of colony formation assay using MMC-3T3 feeder cells pretreated with AdMSC-EVs. (B) Images showing colonies of LECs. MMC-3T3 feeder cells were pretreated with either PBS (control) or AdMSC-EVs (EVs_30 μg/ml and EVs_120 μg/ml). n = 6 biological replicates. (C) The colony-forming efficiency of LECs from the PBS (control) group and EV-treated groups, presented as the mean ± standard deviation (SD). n = 6 biological replicates. (D) Schematic representation of the assessment of MMC-3T3 feeder cells transfected with miRNAs. (E) Images displaying colonies of LECs from the NC group and the miRNA-transfected groups. n = 15 biological replicates. (F) The CFE of LECs from the NC group and miRNA-transfected groups presented as the mean ± SD. n = 15 biological replicates. *p < 0.05, **p < 0.01, and ***p < 0.001. NC: negative control. (PDF) [file pone.0321579.s006.pdf]
