# Supplementary material for: Extracellular vesicles from adipose-derived mesenchymal stem cells promote colony formation ability and EMT of corneal limbal epithelial cells
Source: PLoS One. 2025 Apr 21;20(4):e0321579. doi: 10.1371/journal.pone.0321579 (PMC12011229; doi:10.1371/journal.pone.0321579)
Supplement: Attachment — Submitted filename: Response to Reviewers.docx [file pone.0321579.s008.docx]

Response to Comments

Dear Dr. Mohamed A. M. Ali and reviewers:

Thank you for your comments concerning our manuscript entitled “Extracellular vesicles from adipose-derived mesenchymal stem cells promote colony formation ability and EMT of corneal limbal epithelial cells” (manuscript number: PONE-D-24-38324). The comments were very helpful for revising and improving our paper. We have studied the comments carefully and have made corrections that we hope meet with approval. Revised portions are marked in red within the Revised Manuscript with Track Changes. The main corrections for the paper and responses to each comment are as follows:

Response to the editor’s comments:

1. ***Expand Discussion on Mechanisms****: The role of miRNAs in mediating the observed effects is central to the study, but further elucidation of the exact signaling pathways impacted by miR-25, miR-191, and miR-335 would be valuable. Are these miRNAs directly involved in the regulation of known EMT or stemness-related pathways?*

**Author Response:** Thank you for your suggestions. These miRNAs, we believe, did directly involve in the regulation of EMT-related pathways. *MiR-25-3p* promotes TGF-β-induced EMT in LECs by directly targeting the *SMAD7* gene, thereby downregulating SMAD7 protein that inhibits TGF-β signaling. *MiR-191-5p* may enhance EMT of LECs by inhibiting *KLF6*, a negative regulator of mesenchymal markers. Additionally, *miR-335-5p* might induce EMT by upregulating Ras protein expression. Since it’s known that EMT can induce stemness, we think it’s likely that these miRNAs enhanced the stem cell properties by improving EMT. The above discussions are shown in line 304-316.

2. ***In Vivo Studies****: Consider including in vivo models in future work to validate the therapeutic potential of AdMSC- EVs in a clinical setting. This will enhance the translational relevance of the findings.*

**Author Response:** Thank you for your advice. We’ve done preliminary animal experiments using a rat model to examine the effect of AdMSC-EVs (as shown in the PDF file named "Animal experiments for the editor"). Unfortunately, the first preliminary experiments showed no significant difference between the EV-treated group and the PBS group in the wound healing model. EVs show the effects on corneal epithelial cells *in vitro* when there is sufficient uptake as shown in this study, therefore, this is probably due to insufficient uptake or concentration of EVs *in vivo*. It’s still a problem to determine the optimized concentration of AdMSC-EVs, as well as to improve the uptake of AdMSC-EVs for *in vivo* experiments, which will take more time to solve. In addition, this would suggest that *in vivo* studies require a huge amount of AdMSC-EVs compared to *in vitro* studies. Based on these facts, this study will focus on the effects and mechanism of action of AdMSC-EVs *in vitro*, and as suggested by the editor, we would like to conduct future research on their *in vivo* effects after resolving some of the issues regarding EV administration mentioned above.

3. ***Clarify the Relationship between Proliferation and Colony Formation****: More explanation is needed to reconcile the seemingly paradoxical observation that EVs reduce proliferation while enhancing colony formation. Are there alternative explanations, such as altered cell migration or differentiation status?*
   **Author Response:** Thank you for the valuable suggestions. Various kinds of factors, including cell proliferation, migration, and stemness (differentiation), contribute to colony formation. In this study, we think that enhanced cell migration by AdMSC-EVs particularly increased colony formation. Previous studies (Ref [37-38]) indicated that augmented cell migration increases cell reaggregation, cell splitting and colony splitting, which can increase the number of formed colonies. Furthermore, enhanced stemness via EMT by AdMSC-EVs treatment would also increase colony formation. From the above, it is strongly suggested that the enhancement of cell migration and EMT by EVs comprehensively improved colony formation ability. The above discussion is described in lines 259-269, and 343-352.
4. ***Link EMT with Stemness****: If EMT is driving regenerative properties, the manuscript should make a stronger case for the connection between these two processes. Consider referencing more literature that links EMT with stem cell maintenance, especially in epithelial tissues.*

**Author Response:** Thank you for your helpful comment. We’ve added more sentences and related references (Ref [60-63]) in the discussion part about the connection between EMT and stemness (lines 343-352) according to the editor’s suggestion.

Response to the reviewers’ comments:

*Reviewer #1:*

1. *In methods the authors continuously referred “as previously described” but no reference is added.*

**Author Response:** We are truly sorry for the confusing usage of the phrase. What we meant here is that the methods are as mentioned in this manuscript, not described in previous reports. To avoid confusion, we’ve replaced these phrases with “as described in the section of Colony Formation Assay (lines 472, 476-477, 484-485, and 490-491).

2. *Figure 1, EV 30ug/mL shows statistically significant data than the EV 120 ug/mL. Authors need to clarify this, or need to have more control over experimental data, especially SD.*

**Author Response:** Thank you for your suggestions. Firstly, let us be clear that both EV 30 ug/mL and EV 120 ug/mL significantly increased the CFE of LECs compared to the control group, and there’s no statistically significant difference between EV 30 ug/mL and EV 120 ug/mL. This simply indicates that an EV concentration of 30ug/ml is sufficient and might saturate for its effects on colony formation. In addition, increasing the amount of AdMSC-EVs does not necessarily strengthen its effects because EVs contain diverse biologically active factors, including both positive and negative factors for colony formation or EMT. For instance, it's possible that at high concentrations of AdMSC-EVs, the effects of stemness and EMT may be weakened by increasing the amount of molecules that powerfully suppress EMT or stemness. We’ve added some sentences about the above discussion (lines 293-301).

3. *Figure 2, the data provided don’t support each other. Colony formation is increasing but proliferation is decreased. Colony formation and proliferation are directly related to each other, if colony is increasing its direct indication of proliferation.*

**Author Response:** Thank you for the reviewer’s valuable advice. We agree with the reviewer’s comment that proliferation largely contributes to colony formation. On the other hand, we would like to point out that cells with a high proliferative ability are not necessarily those with a high colony-forming ability. For example, generally, stem cells divide infrequently (so-called "slow cycling cells"), but they exhibit a high colony-forming efficiency. Contrarily, differentiated from stem cells, TA (transient amplifying) cells show higher proliferation than stem cells but low colony-forming efficiency because they rapidly proliferate and are exhausted earlier (Ref [35-36]). As mentioned before, many factors are involved in colony formation, such as cell proliferation, migration, and stemness. Thus, it is difficult to explain the increase or decrease in colony formation due to a single factor, but based on the results of this study, we believe that the enhancement of cell migration and stemness via EMT caused by EVs would contribute to colony formation. We have added these discussions to lines 259-269 and 343-352.

4. *Figure 4, the authors transfected the cell with miR individually, there must be a group where all the miR in cocktail are transfected and then investigate the desired effect.*

**Author Response:** Thank you for your helpful suggestions. We have conducted additional experiments (S5 Fig) using the LECs transfected with either the three most effective miRNA mimics (Mix(3)) or all the miRNA mimics (Mix(6)). The results showed that both transfected groups promoted the colony-forming efficiency compared to the negative control group. And the qRT-PCR revealed that Mix(3) and Mix(6) groups increased expression levels of EMT-related markers *VIM* and *FN1*. Additionally, *deltaNp63* expression levels remained stable, while *KRT12* expression was downregulated in the Mix(3) group and upregulated in the Mix(6) group. Overall, these results showed that the collective effects of these miRNAs on colony formation of LECs were similar to those of the individual miRNA. As mentioned, the mechanism of action of miRNAs in EVs is complex, and in order to maximize their effect, it is likely necessary to optimize the combination of their type, amount, and composition in detail. We have added some sentences about the additional experiment to lines 223-229 and 320-323.

*Reviewer #2:*

1. *Authors are suggested to mention the functional aspect of EVs diversely by briefly mentioning their role in cancer, immunology, etc. Authors are advised to cite these publications. PMID: 36713536, PMID: 36570199.*

**Author Response:** We appreciate your suggestion to cite the references you provided. We have read them and learned a lot. The recommended publications were cited in the revised manuscript [Ref 18-19] (lines 81-82).

2. *Authors are suggested to mention the results only in the result section and the figure legend should be included in the last.*

**Author Response:** Thank you for your mindful suggestions. As the reviewer suggested, we have revised the manuscript to mention experimental results only in the Results section whenever possible. Regarding the figure legends, we confirmed the submission guideline of PLOS ONE, and for figure captions, it says “Each figure caption should appear directly after the paragraph in which they are first cited” (MANUSCRIPT BODY FORMATTING GUIDELINES; <https://journals.plos.org/plosone/s/submission-guidelines>). Thus, following the guidelines, figure legends were left as it is.

3. *The images of electron microscopy 1B, is of low quality. The images of Figure 2 E are also suggested to be replaced by high-quality images.*

**Author Response:** Thanks for the reviewer’s suggestion. We think it is because the upload degraded the image quality when converted to a PDF file. To view the original high-quality TIF image files, you can download them from the links “Click here to access/download;xxx" as shown below.

4. *Figure 4D, images are to be replaced with high quality, nothing is visible in the pdf.* **Author Response:** Thanks for the reviewer's comment. As mentioned above, please download the original TIF image with higher quality. I believe you will then be able to see the details.
5. *The discussion should be shortened.*

**Author Response:** We appreciate the reviewer’s valuable advice. Based on the reviewer’s comments, we have shortened the discussion by removing some sentences related to the results. At the same time, we needed to add some discussion to address the editor’s and reviewer’s feedback. As a result, the overall word count of the revised manuscript has not changed significantly.

6. *Supplementary figures legend should be added in supplementary files only.*

**Author Response:** Thanks for the reviewer's careful reading of our manuscript. We confirmed the submission guideline of PLOS ONE, and for supporting information captions, it says “List Supporting Information captions at the end of the manuscript in a section titled ‘Supporting information’” (MANUSCRIPT BODY FORMATTING GUIDELINES; https://journals.plos.org/plosone/s/submission-guidelines). Thus, following this guideline, we left them as it is.
